# Packing the Punch: Current and Emerging Treatment Strategies in Metastatic Castration-Sensitive Prostate Cancer

**DOI:** 10.1007/s11934-025-01272-6

**Published:** 2025-06-21

**Authors:** Steven P. Troy, Christopher D. Jakubowski, Benjamin A. Gartrell

**Affiliations:** 1 Montefiore Medical Center, Bronx, NY 10467 USA; 2 Montefiore Einstein Comprehensive Cancer Center, Bronx, NY 10461 USA

**Keywords:** metastatic Castration-Sensitive Prostate Cancer (mCSPC), Androgen Deprivation Therapy (ADT), Androgen Receptor Signaling Inhibitor (ARSI), Triplet therapy, Targeted therapies, Personalized treatment

## Abstract

**Purpose of Review:**

Prostate cancer remains a significant source of morbidity and mortality worldwide, with metastatic castration-sensitive disease (mCSPC) representing a complex therapeutic challenge. This review explores how a deeper understanding of the androgen receptor axis has shifted mCSPC management from monotherapy to more intense treatment. We discuss emerging data on triplet regimens, targeted therapies, and the role of local treatment.

**Recent Findings:**

Randomized trials have shown that adding docetaxel or androgen receptor signaling inhibitors (ARSIs) to androgen deprivation therapy (ADT) improves survival. Triplet therapy (ADT, docetaxel, and an ARSI) improves outcomes in patients with high-volume disease compared to ADT and docetaxel alone, although comparisons to ADT plus ARSI doublet therapy are ongoing. The early use of targeted radionuclides, biomarker-driven therapies, and metastasis-directed radiotherapy has also emerged, potentially refining treatment personalization in clinical practice.

**Summary:**

Current guidelines recommend ADT combined with an ARSI, with the addition of docetaxel reserved for high-volume disease. Future research aims to optimize intensity, inform biomarker-driven strategies, and reduce toxicity. Advancements in management of mCSPC underscore the importance of a multimodal, personalized approach to improve outcomes.

**Supplementary Information:**

The online version contains supplementary material available at 10.1007/s11934-025-01272-6.

## Introduction

Prostate cancer is one of the most common solid malignancies among men worldwide and represents a significant cause of morbidity and mortality [1]. Historically, androgen deprivation therapy (ADT) has been the cornerstone of metastatic prostate cancer treatment for over half a century, ever since the foundational studies by Huggins and Hodges in 1941 demonstrated the reliance of prostate cancer cells on androgens for growth and survival [2]. Over the past decade, however, a deeper understanding of prostate cancer biology and the androgen receptor (AR) axis has driven a remarkable transformation in the therapeutic strategies used to treat advanced disease.

While ADT remains a cornerstone of treatment, the management of castration-sensitive metastatic prostate cancer (mCSPC) has become increasingly complex due to the advent of novel therapeutic agents. Up until a decade ago, newly diagnosed metastatic prostate cancer was often treated only with ADT until the disease progressed, at which point second-line treatments were added [3]. However, the results of pivotal trials have demonstrated improved survival with the early addition of chemotherapy or androgen receptor signaling inhibitors (ARSIs) in combination with ADT [3–9].

mCSPC is defined by the presence of metastatic disease coupled with a continued reliance on androgens for tumor growth. It is a setting in which the use of castration is effective in controlling tumor progression; in contrast to castration-resistant prostate cancer (CRPC), which progresses despite adequate testosterone suppression. This review provides a comprehensive overview of the current management strategies of mCSPC, addressing both established treatments and emerging therapeutic approaches.

### Epidemiology and Disease Burden

Prostate cancer ranks among the most frequently diagnosed cancers in men, with an estimated 1.4 million new cases diagnosed globally each year [10]. In the United States, it is the most common cancer in men (excluding non-melanoma skin cancer) and the second leading cause of cancer-related death [11]. Men with metastatic prostate cancer are diagnosed either with de novo (i.e., synchronous) metastatic disease or metastatic recurrence (i.e., metachronous) arising from the progression of localized or locally advanced disease despite prior definitive treatments. Data suggests de novo metastatic disease may have more aggressive disease and inferior outcomes compared to recurrent prostate cancer [12–14].

The extent of metastatic disease can be classified as high- or low-volume based on radiographic findings [3]. High-volume metastatic disease in this setting is commonly defined using the CHAARTED study criteria: four or more bone lesions (with at least one in appendicular skeleton) and/or visceral metastases [3, 15]. This stratification is important because clinical trials have demonstrated that patients with high-volume disease may derive a survival advantage from early intensification of certain systemic therapy options compared to those with low-volume disease [3, 5, 6].

Metastatic prostate cancer spreads to the bones, lymph nodes, and, less frequently, visceral organs such as the liver and lung [16, 17]. Lymph node involvement is categorized as regional or nonregional. Regional lymph nodes include pelvic nodes that directly drain the prostate such as internal and external iliac, obturator, and sacral nodes [18]. Any spread beyond these pelvic lymph nodes, such as to para-aortic, mediastinal, or supraclavicular, is classified as nonregional lymph node metastasis (i.e., distant metastatic disease) [19]. Recognizing this distribution is essential for accurate staging and helps guide decisions for local and systemic treatments.

### Initial Evaluation and Staging

Men newly diagnosed with metastatic prostate cancer should undergo a thorough clinical evaluation and staging workup, which includes detailed history, physical examination, laboratory tests (complete blood count, serum chemistries, serum prostate-specific antigen [PSA], alkaline phosphatase, lactate dehydrogenase, and testosterone levels), imaging studies, and biopsy. Standard imaging modalities typically include technetium-99m bone scans to detect osseous metastases, as well as cross-sectional imaging of soft tissue with computed tomography (CT) of the chest, abdomen, and pelvis [14, 20]. More recently, prostate-specific membrane antigen (PSMA)-based imaging has gained traction for detecting lesions with higher sensitivity and specificity [14, 21, 22]. The biopsy, whether from the primary or a metastatic site, should be performed for pathologic diagnosis and somatic mutation testing [14, 23].

Germline and somatic mutation testing is recommended for all patients with mCSPC due to therapeutic implications, prognostic value, and family risk assessment [14, 24, 25]. Studies have suggested 12–19% of men harbor germline mutations in DNA repair genes such as BRCA2, BRCA1, or others [26, 27]. Although the immediate impact of such findings on the management of mCSPC may be limited at this time, biomarker information may be helpful later in the disease course to identify candidates for poly (ADP-ribose) polymerase (PARP) inhibitors or other targeted therapies [26, 28].

### Traditional Androgen Deprivation Therapy (ADT)

The goal of ADT is to reduce serum testosterone to castration range (< 50 ng/dL) via surgical orchiectomy, gonadotropin-releasing hormone (GnRH) agonists, or GnRH antagonists [29]. While surgical orchiectomy is generally more cost-effective and has similar outcomes compared to chemical castration, it is irreversible and can be psychologically distressing to patients [30]. Most pharmacological ADT agents (e.g., leuprolide, degarelix, goserelin) are administered via subcutaneous or intramuscular injection at regular intervals, however, relugolix is a newer GnRH antagonist which is taken by mouth [31, 32].

ADT as monotherapy, however, is no longer considered sufficient in most patients with newly diagnosed mCSPC. Large, randomized controlled trials (RCTs) have shown that intensification with docetaxel chemotherapy or an ARSI significantly improves outcomes beyond what is achieved with ADT alone [3, 5–8]. Nonetheless, there may be select cases of low-volume, low-risk mCSPC in which ADT alone may be considered, such as patients with limited life expectancy or unwilling to undergo more intensive therapies [14, 33, 34].

Despite its clinical benefits, ADT is associated with multiple adverse effects including hot flashes, loss of libido, erectile dysfunction, metabolic changes (e.g., insulin resistance, weight gain), decreased bone mineral density, and potential cardiovascular complications [35, 36]. To manage hot flashes, pharmacologic interventions (e.g., venlafaxine, oxybutynin, and cyproterone) as well as nonpharmacologic interventions (e.g., acupuncture) may reduce frequency and severity of symptoms [37, 38]. Exercise and dietary interventions can help mitigate weight gain, reduce sarcopenia, and preserve bone mineral density [35, 39]. In patients at high risk of fragility fractures, bone-modifying agents such as bisphosphonates or denosumab may be considered [40–42]. Periodic screening for depression and attention to psychosocial support are indicated for patients on ADT [43]. In addition, GnRH agonists have the potential to cause a disease flare due to the initial testosterone surge [44]. To prevent complications from disease flare, such as spinal cord compression in cases of significant spinal metastases, a first-generation anti-androgen (bicalutamide) should be given while starting a GnRH agonist for at least seven days [44–46].

#### Combining ADT with Chemotherapy (Docetaxel)

A major paradigm shift in the management of mCSPC occurred when clinical trials reported that the early addition of docetaxel to ADT improved survival in men with metastatic disease, particularly those with high-volume disease [3, 5]. Docetaxel is a taxane chemotherapy agent that stabilizes microtubules and leads to cell cycle arrest and apoptosis [47]. It had previously been demonstrated to improve survival in men with mCRPC [48]. Its role in the castration-sensitive setting was explored by several key studies:

##### CHAARTED (E3805)

In the CHAARTED trial, men with newly diagnosed mCSPC were randomized to ADT plus docetaxel or ADT alone. Patients were prospectively risk-stratified by high- or low-volume disease [3]. The primary endpoint was overall survival (OS). Median OS was 57.6 months in the ADT plus docetaxel group and 44 months in the ADT alone group (hazard ratio [HR] 0.61, *P* < 0.001) [3]. The survival benefit was particularly notable in the subgroup with high-volume disease; the median OS in this subgroup was 49.2 months in patients receiving docetaxel plus ADT versus 32.2 months in patients receiving ADT alone (HR 0.60, *P* < 0.001) [3].

##### STAMPEDE

The STAMPEDE trial is a multi-arm, multi-stage platform study evaluating various strategies in men with high-risk locally advanced, recurrent, or metastatic prostate cancer [49]. Arm C compared docetaxel plus standard of care (SOC) with SOC alone in patients with mCSPC. The primary endpoint was median OS. The median OS was 60 months for patients with mCSPC given docetaxel plus SOC compared to 45 months in patients given SOC alone (HR 0.76, *P* = 0.005) [4, 50]. The survival benefit from adding docetaxel was seen in both low-volume and high-volume disease [50].

##### GETUG-AFU15

In this phase III open-label study, patients with newly diagnosed mCSPC were assigned to either docetaxel plus ADT or ADT alone [51]. Median OS in patients in the docetaxel plus ADT group was 62.1 months versus 48.6 months in the group treated with ADT alone (HR 0.88, *p* = 0.3), thus demonstrating docetaxel had no significant improvement in OS [52]. A post-hoc analysis stratifying patients into high- and low-volume groups according to CHAARTED trial criteria also showed no significant OS benefit from docetaxel in either subgroup [52]. When data from this study was aggregated with CHAARTED trial data in a meta-analysis, however, docetaxel plus ADT improved OS compared to ADT alone in patients with high-volume disease (51.2 months versus 39.8 months; HR 0.68, *p* < 0.001) [53].

The conclusions of these trials and analyses prompted guideline committees at the time to endorse ADT plus docetaxel as a standard first-line treatment option for men with newly diagnosed high-volume mCSPC [15, 54]. The use of docetaxel, however, was limited due its toxicities such as myelosuppression, neuropathy, and fatigue [47, 55]. Moreover, following further recent advancements described below, ADT plus docetaxel alone became a less favored first-line treatment option for mCSPC.

### Adding Androgen Receptor Axis-Targeted Therapies

While docetaxel chemotherapy improved outcomes significantly, further breakthroughs came with the introduction of potent androgen receptor pathway inhibitors (ARSIs), specifically abiraterone acetate, enzalutamide, apalutamide, and darolutamide which have each demonstrated survival benefits when added to ADT in the mCSPC setting [5, 7, 8, 56, 57]. Unlike docetaxel, these agents specifically target the AR signaling axis, and are generally better tolerated compared to docetaxel, although each has a unique side-effect profile [58].

#### Abiraterone Acetate

Abiraterone acetate is a prodrug of abiraterone, which irreversibly inhibits CYP17A1, an enzyme critical to androgen biosynthesis in the testes, adrenal glands, and within the tumor microenvironment [59]. By blocking CYP17A1, abiraterone reduces testosterone production to extremely low levels, enhancing the suppression already achieved by ADT and targeting residual androgen sources that can drive tumor growth [60]. The role of abiraterone in mCSPC was established by two landmark studies.

##### LATITUDE

This phase III double-blind trial recruited men with newly diagnosed, high-risk mCSPC defined by at least two of the following: Gleason score ≥ 8, presence of three or more bone lesions, or measurable visceral metastases. Patients were randomized to receive ADT plus abiraterone and low-dose prednisone versus ADT plus placebo [6]. OS and radiographic progression-free survival (rPFS) were primary endpoints. Median OS in the group receiving ADT plus abiraterone and low-dose prednisone was 53.3 months versus 36.6 months in ADT plus placebo (HR 0.66, * P* < 0.0001). The median rPFS was 33 months in the combination group versus 14.8 months in the placebo group (HR 0.47, *P* < 0.001) [61].

##### STAMPEDE

Arm G of the STAMPEDE trial evaluated ADT plus abiraterone with low-dose prednisone versus SOC in men with locally advanced or metastatic prostate cancer. The primary endpoint of the study was OS [5]. For patients with metastatic disease, median OS was 76.6 months in patients receiving abiraterone versus 45.7 months in patients receiving SOC (HR 0.62, *P * < 0.0001) [62].

Based on the results of LATITUDE and STAMPEDE, abiraterone plus ADT became a first-line option for men with newly diagnosed mCSPC [63].

Common toxicities of abiraterone include hypertension, hypokalemia, and fluid retention, reflecting the systemic effects of CYP17A1 inhibition on steroid metabolism [59]. Hepatotoxicity is another known side effect [64]. Patients receiving abiraterone require close monitoring of blood pressure, serum electrolytes (especially potassium), and liver function tests [65]. The co-administration of low-dose steroids helps mitigate mineralocorticoid-related toxicities such as hypokalemia and fluid overload, but side effects from chronic low-dose steroids such as hyperglycemia should be considered [66, 67]. Cardiovascular assessment is also important, given the risk of exacerbating underlying hypertension [68]. In certain patients with significant cardiovascular disease or uncontrolled diabetes mellitus, alternative treatment options should be considered [69, 70].

#### Enzalutamide

Enzalutamide is a second-generation nonsteroidal anti-androgen that blocks multiple steps of AR signaling, including AR binding to androgens, nuclear translocation of the AR, and AR binding to DNA [71]. The efficacy of enzalutamide was highlighted by two seminal trials: ENZAMET and ARCHES.

##### ENZAMET

In the open-label phase III ENZAMET trial, men with mCSPC were randomized to receive either enzalutamide plus ADT or a first-generation anti-androgen plus ADT, with or without early docetaxel [7]. The primary endpoint was OS. Over a median follow up of 34 months, the HR of death in patients treated with enzalutamide compared to a standard nonsteroidal antiandrogen was 0.67 (*P* = 0.002) [7]. Although the study lacked statistical power to draw reliable conclusions from the early docetaxel subgroup, patients in this subgroup experienced more adverse effects and had less OS benefit (HR 0.90, 95% confidence interval [CI] 0.62–1.31) compared to those who did not receive early docetaxel (HR 0.53, 95% CI 0.37–0.75).

##### ARCHES

The ARCHES trial was a phase III, double-blind RCT which evaluated enzalutamide plus ADT versus placebo plus ADT in mCSPC. The primary endpoint was rPFS. Enzalutamide demonstrated a significant improvement in rPFS; the HR of rPFS after median follow up of 14.4 months was 0.39 (*P* < 0.001) [72]. The impressive improvement of rPFS in this study led to the approval of enzalutamide in combination with ADT [73]. Subsequent analysis with complete data confirmed OS benefit (HR 0.66, 95% CI 0.53–0.81) [73].

Enzalutamide can cause fatigue, hypertension, and rarely seizures [71]. Avoiding enzalutamide in patients with a history of seizure should be considered. Monitoring of neurocognitive side effects and potential interactions with other medications metabolized by CYP enzymes is also warranted [74, 75].

#### Apalutamide

Apalutamide is another second-generation AR antagonist that binds directly to the ligand-binding domain of the AR, inhibiting AR nuclear translocation, DNA binding, and AR-mediated transcription [76]. Apalutamide’s role in mCSPC was evaluated in the TITAN trial.

##### TITAN

This phase III trial compared apalutamide plus ADT to placebo plus ADT in men with mCSPC. The two primary endpoints were rPFS and OS. After a median of 22.7 months of follow up, patients assigned to ADT plus apalutamide had an improved rPFS and OS compared to ADT plus placebo; rPFS HR was 0.48 (*P* < 0.001) and OS HR was 0.67 (*P* = 0.005) [8]. Given the disparity in outcomes, patients were unblinded and crossed over to receive apalutamide. A final analysis following the crossover calculated OS HR of 0.52 (*P* < 0.0001) [77]. Based on the results of this large RCT, apalutamide became an additional standard option for men with newly diagnosed mCSPC.

Adverse effects of apalutamide include fatigue, hypertension, and potential increased risk of falls/fractures [78]. There is also a rare risk of seizure which was reported in 0.4% of patients taking apalutamide in the TITAN trial; apalutamide should be avoided in patients with a history of seizures [8, 78]. A unique side effect associated with apalutamide is a higher incidence of rash [78]. Dose modifications or interruptions may be required in cases of severe rash [79].

#### Darolutamide

Another AR pathway inhibitor, darolutamide, has also shown promise in both the mCRPC and the mCSPC setting. Its efficacy in mCSPC was highlighted in the ARASENS and ARANOTE trials.

##### ARASENS

The ARASENS trial was a phase III double-blind RCT which was designed to compare the combination of darolutamide plus ADT and docetaxel to placebo plus ADT and docetaxel in men with mCSPC. The primary endpoint was OS. The median OS in patients who received darolutamide plus ADT and docetaxel was not reached compared to 48.9 months in patients who received placebo plus ADT and docetaxel (HR 0.68, *P* < 0.001) [9].

##### ARANOTE

Building on the positive findings from ARASENS, the phase III ARANOTE trial evaluated darolutamide plus ADT versus placebo plus ADT in men with mCSPC who were not receiving up-front docetaxel. The primary endpoint was rPFS. At time of first analysis, rPFS in patients receiving darolutamide was not reached versus 25.0 months in patients receiving placebo (HR 0.54, *P* < 0.0001); OS data has not yet matured [57].

Both ARASENS and ARANOTE trials, as well as meta-analyses of ARSIs in non-metastatic prostate cancer and mCRPC, suggest that darolutamide has fewer and less severe adverse effects compared to enzalutamide and apalutamide [9, 57, 80–82]. The molecular structure of darolutamide inherently reduces its penetration through the blood-brain barrier compared to other ARSIs, thus the risk of central nervous system adverse effects such as seizure are felt to be lower [83, 84].

### Triplet Therapy and the Current Standard

The combination of ADT, docetaxel, and ARSI (i.e., triplet therapy) has been compared to ADT plus docetaxel (i.e., doublet therapy) in several studies. The ARASENS trial showed that adding darolutamide to ADT plus docetaxel yielded a significant OS improvement compared to placebo plus ADT plus docetaxel in patients with mCSPC [9]. Additionally, the PEACE-1 trial found that the combination of abiraterone plus ADT plus docetaxel improved rPFS and OS compared to ADT plus docetaxel in men with de novo mCSPC [85]. A network meta-analysis using data from ten trials found that triplet therapies of darolutamide and abiraterone had better OS compared to doublet therapies, however, the difference was not statistically significant [86]. It is important to note that, in large RCTs to date investigating the efficacy of triplet therapy, the control arms have been doublets with docetaxel rather than an ARSI. Thus, triplet therapy has not yet been compared to a doublet containing an ARSI. An ongoing phase II randomized trial, NCT06060587, is comparing triplet therapy to ADT plus abiraterone; results of this trial, as well as other future studies, will help clarify the benefit of triplet therapy compared to a doublet containing an ARSI [87].

In summary, for newly diagnosed mCSPC, the standard of care typically includes treatment with ADT plus an ARSI (e.g., abiraterone, enzalutamide, apalutamide, darolutamide). The decision to add docetaxel depends on disease volume (high versus low) and other clinical factors. In high-volume disease, combining ADT with both an ARSI and docetaxel may offer better disease control, whereas for low-volume disease, docetaxel is usually not necessary.

### Role of Local Treatments to the Prostate in mCSPC

Historically, once prostate cancer had metastasized, local radiation therapy to the prostate or metastatic sites was primarily used for palliation, typically aimed only at mitigating local complications or painful metastases [88]. However, growing evidence suggests that local consolidative radiotherapy to the primary tumor can improve outcomes in selected patients with de novo mCSPC [89].

Arm H of the STAMPEDE trial evaluated the addition of external-beam radiotherapy to the prostate in men with newly diagnosed mCSPC [90]. Although no significant OS benefit was seen in the study population as a whole, secondary analysis revealed that patients with low metastatic burden had improved OS (HR 0.62, 95% CI 0.46–0.83) [90, 91]. Two other clinical trials, HORRAD and PEACE-1, also evaluated efficacy of radiotherapy to primary tumor in patients with mCSPC. Both of these trials demonstrated PFS benefit without OS benefit in men with low-volume mCSPC [92, 93]. A meta-analysis, STOPCAP, pooled data from STAMPEDE and HORRAD and concluded OS benefit in men with four or less bone metastases [94]. Guidelines now support considering prostate radiotherapy for men with low-volume disease [54]. Ongoing research aims to refine patient selection and evaluate whether radical prostatectomy might be beneficial for certain subsets with metastatic disease [95].

### Emerging Approaches and Future Directions

Despite major therapeutic advances in mCSPC, disease progression remains inevitable. Ongoing research continues to explore novel strategies that might further delay progression, prolong survival, and improve quality of life.

#### Biomarker-Driven Therapies and Genomics

Advances in molecular characterization of prostate cancer have allowed identification of mutations in DNA damage repair genes which may be targeted with PARP inhibitors or other targeted therapies [28, 96–101]. Two ongoing phase III RCTs, AMPLITUDE and EvoPAR-Prostate01, are evaluating the efficacy of PARP inhibitors niraparib and saruparib, respectively, in combination with ADT and an ARSI in patients with mCSPC [102, 103]. Another potentially targetable pathway in mCSPC is phosphatase and tensin homolog (PTEN)-phosphatidylinositol 3-kinase (PI3K)-AKT [104]. Capivasertib is a selective pan-AKT inhibitor being studied in the phase III CAPItello-281 trial. This study is comparing outcomes in patients who have synchronous mCSPC with PTEN-deficient tumors when treated with capivasertib plus abiraterone plus ADT versus placebo plus abiraterone plus ADT [105]. Early results published via press release showed improved rPFS in patients assigned to capivasertib group compared to placebo group [106].

#### Metastasis-Directed Therapy

Metastasis-directed therapy (MDT) is stereotactic body radiotherapy (SBRT) directed at metastases. In the case of mCSPC, MDT has been investigated as an alternative to observation in patients with oligometastatic, metachronous prostate cancer. The STOMP trial, a phase II RCT, was designed to compare MDT to observation in patients with metachronous mCSPC who had less than four metastases [107]. Median PFS was 13 months for patients in the observation group versus 21 months in patients in the MDT group (HR 0.60, log rank *P* = 0.11) [107]. A subsequent phase II RCT, the ORIOLE trial, also compared the efficacy of MDT versus observation in patients with low-volume metachronous mCSPC, and found median PFS was not reached in MDT group opposed to 5.8 months in observation group (HR 0.030, *P* = 0.002) [108]. Meta-analysis of combining data of both studies showed MDT provided a sustained clinical benefit compared to observation (HR 0.44, *P* < 0.001) [109]. Results of ongoing RCTs will help refine the role of MDT in oligometastatic mCSPC [110, 111].

#### PSMA-Targeted Approaches

PSMA-targeted radionuclides such as ^177^Lu-PSMA-617 are being investigated as a treatment option for mCSPC as they have shown efficacy in mCRPC patients who have had progression on ARSI and taxane-based chemotherapy [112–114]. UpFrontPSMA, a phase II RCT, showed ^177^Lu-PSMA-617 plus docetaxel increased tumor response in patients with high-volume mCSPC compared to docetaxel alone [115]. An ongoing phase III RCT, PSMAddition, is comparing outcomes of ^177^Lu-PSMA-617 plus SOC versus SOC alone in patients with treatment-naïve mCSPC [116].

#### Therapy De-Escalation

With an expanding menu of effective agents, critical questions arise regarding appropriateness of de-escalating therapy. De-escalation is an important area of study considering the potential benefits of reduced long-term toxicity and costs. One of the first large RCTs that studied de-escalation in mCSPC was SWOG S9346 (INT-0162), which compared intermittent ADT to continuous ADT in patients with mCSPC when PSA fell to less than 4 ng per milliliter [117]. The study found that intermittent ADT did not meet the predefined criteria for noninferiority in OS (HR 1.10; 90% CI 0.99–1.23), although there were statistically significant improvements in side effects including erectile dysfunction and mental health [117]. The phase II trial A-DREAM/Alliance A032101 is currently evaluating the impact of interrupting doublet therapy in patients with mCSPC who have demonstrated an excellent response to long-term treatment [118, 119]. As treatments for mCSPC advance, the goal will be to find the most efficacious and effective regimens without unnecessary toxicity or costs.

## Conclusion

Over the past decade, the management of mCSPC has evolved from single-agent ADT to a paradigm of doublet and triplet therapies. Large clinical trials first demonstrated that early intensification by adding docetaxel to an ADT backbone improved outcomes for patients with high-volume disease. Subsequent clinical trials added ARSIs to existing treatment regimens, and results showed remarkable efficacy when combined into doublet or triplet therapies. In clinical practice, the current standard of care for mCSPC is ADT plus an ARSI with docetaxel considered for high-volume disease. Despite significant strides made over the past decade, important questions remain. One critical debate persists about whether a triplet regimen outperforms doublet therapy with an ARSI.

There are several emerging areas of investigation in mCSPC therapies which may further improve outcomes. Trials evaluating targeted treatments such as PARP inhibitors and AKT inhibitors are ongoing, potentially offering a personalized approach for patients with corresponding biomarkers. Further investigation into the use of metastasis-directed therapy and PSMA-targeted radioligand therapy may also expand the repertoire of first-line treatment options. These evolving strategies underscore the continued need for an approach that customizes therapy to individual patients based on disease biology and clinical factors.

## Key References

- Saad F, Vjaters E, Shore N, et al. (2024) Darolutamide in Combination With Androgen-Deprivation Therapy in Patients With Metastatic Hormone-Sensitive Prostate Cancer From the Phase III ARANOTE Trial. J Clin Oncol 42:4271–428.
-
  - Provides phase III data showing the efficacy and safety of darolutamide in combination with ADT (without docetaxel) for mCSPC, suggesting an additional treatment option for patients who may not be candidates for chemotherapy.
- Fizazi K, Foulon S, Carles J, et al. (2022) Abiraterone plus prednisone added to androgen deprivation therapy and docetaxel in de novo metastatic castration-sensitive prostate cancer (PEACE-1): a multicentre, open-label, randomised, phase 3 study with a 2 × 2 factorial design. The Lancet 399:1695–1707.
-
  - Establishes the potential benefit of a triplet strategy (abiraterone, docetaxel, and ADT) in patients with newly diagnosed, high-burden mCSPC.
- Mandel P, Hoeh B, Wenzel M, Preisser F, Tian Z, Tilki D, Steuber T, Karakiewicz PI, Chun FKH (2023) Triplet or Doublet Therapy in Metastatic Hormone-sensitive Prostate Cancer Patients: A Systematic Review and Network Meta-analysis. Eur Urol Focus 9:96–105.
-
  - Summarizes and compares multiple trials of doublet versus triplet regimens, highlighting the ongoing debate over whether three-agent approaches provide a clinically meaningful advantage over two-agent combinations.
- Azad AA, Bressel M, Tan H, et al. (2024) Sequential [177Lu]Lu-PSMA-617 and docetaxel versus docetaxel in patients with metastatic hormone-sensitive prostate cancer (UpFrontPSMA): a multicentre, open-label, randomised, phase 2 study. Lancet Oncol 25:1267–1276.
-
  - Demonstrates the efficacy of PSMA-targeted radionuclide therapy as part of first-line management of high-volume mCSPC. The data suggest that adding [177Lu]Lu-PSMA-617 to docetaxel intensifies tumor response, potentially changing the treatment landscape if confirmed in larger phase III trials.

## Electronic supplementary material

Below is the link to the electronic supplementary material.

Supplementary Material 1

Supplementary Material 2

## Data Availability

No datasets were generated or analysed during the current study.
